# Fresh aboveground net primary productivity of Tibetan grasslands: Responses of different plant functional groups to climate change and human activities and implications for ecosystem management

**DOI:** 10.1371/journal.pone.0349705

**Published:** 2026-06-10

**Authors:** Guangyu Zhang, Wei Sun, Shaowei Li, Zhiming Zhong, Yuan Tian, Chenjun Zhao, Fusong Han, Shaolin Huang, Dunzhu Yujie, Gang Fu

**Affiliations:** 1 Lhasa Plateau Ecosystem Research Station, Key Laboratory of Ecosystem Network Observation and Modeling, Institute of Geographic Sciences and Natural Resources Research, Chinese Academy of Sciences, Beijing, China; 2 College of Resources and Environment, University of Chinese Academy of Sciences, Beijing, China; 3 College of Urban and Environmental Sciences, Hunan University of Technology, Zhuzhou, Hunan, China; 4 College of Tourism, Henan Normal University, Xinxiang, Henan, China; 5 People’s Government of Zhongba County, Xigaze, Tibet Autonomous Region, China; University of Udine: Universita degli Studi di Udine, ITALY

## Abstract

Uncertainties remain regarding how climate change and human activities affect the aboveground net primary productivity (ANPP) of grassland ecosystems, particularly the differential responses of distinct plant functional groups. Here, we investigate the alpine grasslands of the Qinghai-Xizang Plateau from 2000 to 2022, focusing on the fresh ANPP of the whole plant community and three key functional groups (sedges, graminoids, and forbs). Our objectives are to identify the spatiotemporal trends of ANPP and to determine the dominant drivers (climate vs. human activities) of these changes. Under the combined effects of climate change and human activities, the spatially averaged relative changes in fresh ANPP were –1.15% for the whole community, + 5.67% for sedges, –1.55% for graminoids, and –2.26% for forbs. Regional patterns varied, with some areas showing increasing or decreasing trends over time, while others exhibited no significant change. Climate change dominated 20.79% of the grassland area, human activities dominated 54.26%, and the two drivers jointly dominated 24.95%. When the ANPP of the three functional groups were considered together, the area where all three groups jointly regulated grassland fresh ANPP accounted for the largest proportion (39.12%), followed by areas dominated by forbs (35.43%), sedges (18.27%), and graminoids (7.18%). These results reveal pronounced geographical heterogeneity in the trends of fresh ANPP, both for the whole community and for individual functional groups. Human activities exert a larger controlling influence than climate change over the observed ANPP changes. Moreover, the contribution of each functional group to community ANPP varies spatially. Our findings provide a scientific basis for understanding grassland ecosystem functioning and for developing targeted conservation and management strategies.

## Introduction

In the global ecosystem, the research on the response of aboveground net primary productivity (ANPP) of grassland ecosystems to climate change and human activities is of great significance. Many studies have widely and deeply explored the relationships between ANPP and climate change and human activities in different ecosystems and have achieved fruitful results [1,2]. These studies provide a crucial basis for understanding the functions and dynamic changes of ecosystems and promote the development of the field of ecology. Three key scientific questions remain insufficiently addressed. First, while the dynamic responses of ANPP are a core topic in global change studies, existing studies have mostly focused on the responses of the dry ANPP to climate change and human activities, with insufficient attention paid to fresh ANPP [3,4]. As a key indicator directly reflecting plant water status, immediate metabolic activity, and forage availability, the responses of fresh ANPP to climate change and human activities have not yet formed a systematic understanding. For example, the spatiotemporal dynamic patterns of fresh ANPP still lack clear answers. Second, the impact of climate change and human activities on the ANPP of plant community has been mostly quantified [5,6]. However, on a large spatial scale, the research on the impact of ANPP of different plant functional groups (e.g., sedges and graminoids) is lacking [4,7]. Third, the main focus has been on the direction of ANPP change (increase, decrease or no change). However, relatively little attention has been paid to whether this increasing or decreasing trend is gradually intensifying or weakening. In general, although there are many research results related to ANPP of global grassland ecosystems, the above-mentioned problems still need to be further explored in order to accurately understand the functions and dynamic change mechanisms of grassland ecosystems.

The Qinghai-Xizang Plateau’s alpine grasslands are globally unique and highly sensitive to climate change, distinct from other grassland systems. As the “Roof of the World” and “Third Pole,” its average elevation exceeds 4,000 m, creating extreme topography, shaping a climate with low temperatures, strong radiation, and large diurnal variations. It acts as a critical “climate regulator,” influencing East Asian monsoons and global climate, making its grasslands key in biosphere-atmosphere interactions. Ecologically fragile yet biodiverse, these grasslands support endemic species adapted to harsh conditions, serve as major carbon sinks, and conserve water for rivers sustaining billions. However, low productivity and slow nutrient cycling make them vulnerable to disturbances [8]. Notably, the Qinghai-Xizang Plateau warms twice as fast as the global average, with altered precipitation and radiation [9]. These changes disproportionately affect its grasslands, causing amplified, nonlinear responses. Studying their fresh ANPP dynamics is vital for understanding regional resilience and high-altitude ecosystem responses to global change [10]. In the alpine grasslands of the Qinghai-Xizang Plateau, the ecological significance of the fresh ANPP is particularly prominent. First, fresh ANPP directly determines the palatability and nutritional value of forage, which is crucial for the sustainable development of grazing animal husbandry. Its dynamic fluctuations directly affect the feeding efficiency and health status of livestock. Second, in alpine regions with drastic day-night temperature differences and frequent precipitation, the response sensitivity of plant fresh ANPP to short-term changes in water and heat is much higher than that of dry ANPP, and it can more quickly and accurately reflect the immediate functional state of the ecosystem. Therefore, focusing on the response patterns of fresh ANPP is a key entry point for analyzing the correlation between the ecological functions of alpine grasslands and the sustainability of animal husbandry.

The unique sensitivity of the Qinghai-Xizang Plateau’s alpine grasslands makes understanding their ANPP responses critical. While existing studies using in-situ experiments and models have advanced knowledge of dry ANPP dynamics in alpine grasslands [3,11], which is beneficial for understanding the evolution of the alpine ecosystems. Two further uncertainties remain. First, the dominant functional group (sedges, graminoids, or forbs) driving plant community ANPP changes are still unresolved [4,11]. Second, the influence of changes in total shortwave radiation (a crucial component of surface energy balance affecting evapotranspiration and photosynthetic potential) on ANPP has received comparatively less attention than warming or precipitation changes. Addressing these uncertainties is essential for a comprehensive understanding of alpine grassland ecosystem functioning under global change. Therefore, further exploration is needed to comprehensively and accurately understand the operation mechanism and development trend of the alpine grassland ecosystems in this area.

In view of this background, this study was based on the modelled data of the fresh ANPP of plant community and different functional groups (sedges, graminoids and forbs), soil carbon, nitrogen and phosphorus of alpine grasslands on the Qinghai-Xizang Plateau from 2000 to 2022. It statistically analyzed the change characteristics of the data under the combined and individual impacts of climate change and human activities, used structural equation models to quantify the impact of various factors on the change of fresh ANPP, and deeply explored the dominant factors and their geographical variability of the changes of fresh ANPP of different plant functional groups and plant community. The main aim was to comprehensively reveal the change and driving mechanisms of fresh ANPP of this alpine grassland ecosystems, provide theoretical support for formulating ecological protection and management strategies, respond to the challenges of climate change and human activities, and maintain its sustainable development.

## Materials and methods

### Data

In previous studies [12–15], we constructed random forest models for two scenarios: (1) using observed temperature, precipitation, and shortwave radiation as driving factors to simulate fresh ANPP, soil organic carbon, total nitrogen, total phosphorus, carbon-nitrogen ratio, carbon-phosphorus ratio, nitrogen-phosphorus ratio, pH, ammonium nitrogen, nitrate nitrogen, and available phosphorus of plant communities and functional groups for the climate‑only scenario (fenced conditions); (2) based on the abovementioned climatic factors, the maximum normalized difference vegetation index was added as an input variable to simulate the same ecological and soil indicators for the combined effect scenario of climate change and human activities (grazing conditions). All the random forest models were validated for accuracy using at least 30 sets of independent observation data, with verification indicators including relative bias and root mean square error. The absolute values of the relative bias of these models were all less than 9.00% [8,16]. Based on these previously constructed random forest models of fresh ANPP of plant community, sedges, graminoids, and forbs, and soil carbon, nitrogen, phosphorus, and pH and their derived soil data (soil organic carbon, SOC; total nitrogen, TN; total phosphorus, TP; ammonium nitrogen, NH_4_^+^-N; nitrate nitrogen, NO_3_^-^-N; available phosphorus, SAP, pH, ratio of SOC to TN, C:N; ratio of SOC to TP, C:P; and ratio of TN to TP, N:P) [12–15], we obtained the raster data of the concerned variables over the entire alpine grasslands on the Qinghai-Xizang Plateau for each year from 2000 to 2022 under the combined effects of climate change and human activities, and the climate‑only effect, respectively. Referring to the studies of predecessors [17,18], the ratio of the soil carbon, nitrogen, phosphorus and pH and their derived data under the combined influence of climate change and human activities to those under climate‑only influence was taken as the soil carbon, nitrogen, phosphorus and pH and their derived data of the alpine grasslands across the Qinghai-Xizang Plateau from 2000 to 2022 under the human‑activity‑only influence, respectively. Referring to previous studies [19], the difference between fresh ANPP of plant community (ANPP_C_), sedges (ANPP_S_), graminoids (ANPP_G_) and forbs (ANPP_F_) under climate‑only scenario and that under the combined influence of climate change and human activities was regarded as the fresh ANPP of plant community, sedges, graminoids and forbs under the human‑activity‑only scenario, respectively. The fresh ANPP_C_C+H_, ANPP_S_C+H_, ANPP_G_C+H_ and ANPP_F_C+H_ indicated the fresh ANPP_C_, ANPP_S_, ANPP_G_ and ANPP_F_ under the combined effects of climate change and human activities, respectively. The fresh ANPP_C_C_, ANPP_S_C_, ANPP_G_C_ and ANPP_F_C_ indicated the fresh ANPP_C_, ANPP_S_, ANPP_G_ and ANPP_F_ under the climate‑only effect, respectively. The fresh ANPP_C_H_, ANPP_S_H_, ANPP_G_H_ and ANPP_F_H_ indicated the fresh ANPP_C_, ANPP_S_, ANPP_G_ and ANPP_F_ under the human‑activity‑only effect, respectively. The abbreviations of soil variables were similar to those of fresh ANPP of plants.

Annual temperature (AT), annual precipitation (AP) and annual radiation (ARad) data from 2000 to 2022 were derived from the previously published raster datasets of monthly average temperature, monthly precipitation and monthly radiation, respectively [20,21]. The annual maximum normalized difference vegetation index (NDVI_max_) data was derived from the normalized difference vegetation index data product of the Moderate Resolution Imaging Spectroradiometer (MODIS). The temporal and spatial resolutions of this product are one month and one kilometer, respectively.

### Statistical analyses

Referring to previous studies [22,23], we calculated the change rate of AT (ΔAT), AP (ΔAP), ARad (ΔARad) and NDVI_max_ (ΔNDVI_max_) for all pixels over the alpine grasslands on the Qinghai-Xizang Plateau from 2000 to 2022. Similarly, we also calculated the change rate of the fresh ANPP of plants and soil variables for all pixels over same region and time period. Following previous studies [22,23], this study calculated the raster dataset of the relative change of fresh ANPP of plant community, sedges, graminoids and forbs, and soil data, over the period 2000–2022, respectively. For example, first, we obtained the change rate of the fresh ANPP of plant community under the combined influence of climate change and human activities (ΔANPP_C_C+H_) for the period 2000–2022 using the sens.slope function of ‘trend’ package. Then, we multiplied ΔANPP_C_C+H_ by 22 and divided the result by the fresh ANPP of plant community in 2000 to get the relative change of ANPP_C_C+H_ (RC_ANPP_C_C+H_). The abbreviations of all other relative changes were similar to RC_ANPP_C_C+H_. In addition to quantifying the overall change rate of the fresh ANPP from 2000 to 2022, this study also quantified the change rate of the change rate (i.e., to reflect whether the change rate of a variable was increasing or decreasing). The relevant steps were detailed below taking the fresh ANPP of plant community under the combined effects of climate change and human activities (ANPP_C_C+H_) as an example. First, the change rates of the fresh ANPP of plant community from 2000 to 2022 were calculated using a sliding window (window size = 3). Thus, 19 change rates of this variable were obtained. Then, based on the above 19 change rates, their change rate, that is, the change rate of the change rate (ΔΔANPP_C_C+H_), was calculated. If the overall change rate of this variable from 2000 to 2022 was > 0, and the change rate of the change rate was also > 0, it indicated that the positive response of this variable was strengthening year by year (**Table 1**). If the overall change rate of this variable from 2000 to 2022 was < 0, but the change rate of the change rate was > 0, it indicated that the negative response of this variable was weakened year by year (**Table 1**). This study quantified the dominance of the fresh ANPP of sedges, graminoids and forbs over that of plant community (**Table 2**). Referring to previous studies [19,22], we quantified the relative impacts of climate change and human activities to the fresh ANPP of plant community, sedges, graminoids, and forbs. Finally, the structural equation models were utilized to quantify the influence of independent variables on the changes in the fresh ANPP of plants. All statistical analyses were completed using R software (version 4.2.2).

**Table 1:** Classification of the change patterns of fresh aboveground net primary productivity of the plant community under combined climate and human influences (ANPP_C_C+H_).

ΔANPP_C_C + H_	ΔΔANPP_C_C + H_	Changes
>0	>0	Increases in positive response of ANPP_C_C + H_
>0	<0	Decreases in positive response of ANPP_C_C + H_
>0	=0	No change in positive response of ANPP_C_C + H_
<0	>0	Decreases in negative response of ANPP_C_C + H_
<0	<0	Increases in negative response of ANPP_C_C + H_
<0	=0	No change in negative response of ANPP_C_C + H_
=0	–	No change of ANPP_C_C + H_

**Note:** ΔANPP_C_C+H_, change rate of ANPP_C_C+H_. ΔΔANPP_C_C+H_, change in ΔANPP_C_C+H_ (i.e., acceleration or deceleration of the trend). > 0, < 0, = 0 indicate positive, negative, or zero values, respectively. A dash (–) indicates that the value is not applicable because ΔANPP_C_C+H_ = 0.

**Table 2:** Relative contributions of three plant functional groups (sedges, graminoids, and forbs) to the change in fresh aboveground net primary productivity of the plant community under combined climate and human influences.

ΔANPP_C_C + H_	Comparison of ANPP_S_C + H_, ANPP_G_C + H_ and ANPP_F_C + H_	Causes
ΔANPP_C_C + H_ > 0	|ΔANPP_S_C + H_| > |ΔANPP_G_C + H_| & |ΔANPP_S_C + H_| > |ΔANPP_F_C + H_|	The increase of ANPP_C_C + H_ due to sedges
ΔANPP_C_C + H_ > 0	|ΔANPP_G_C + H_| > |ΔANPP_S_C + H_| & |ΔANPP_G_C + H_| > |ΔANPP_F_C + H_|	The increase of ANPP_C_C + H_ due to graminoids
ΔANPP_C_C + H_ > 0	|ΔANPP_F_C + H_| > |ΔANPP_S_C + H_| & |ΔANPP_F_C + H_| > |ΔANPP_G_C + H_|	The increase of ANPP_C_C + H_ due to forbs
ΔANPP_C_C + H_ > 0	|ΔANPP_S_C + H_| = |ΔANPP_G_C + H_| & |ΔANPP_S_C + H_| = |ΔANPP_F_C + H_|	The increase of ANPP_C_C + H_ due to all three groups equally
ΔANPP_C_C + H_ < 0	|ΔANPP_S_C + H_| > |ΔANPP_G_C + H_| & |ΔANPP_S_C + H_| > |ΔANPP_F_C + H_|	The decrease of ANPP_C_C + H_ due to sedges
ΔANPP_C_C + H_ < 0	|ΔANPP_G_C + H_| > |ΔANPP_S_C + H_| & |ΔANPP_G_C + H_| > |ΔANPP_F_C + H_|	The decrease of ANPP_C_C + H_ due to graminoids
ΔANPP_C_C + H_ < 0	|ΔANPP_F_C + H_| > |ΔANPP_S_C + H_| & |ΔANPP_F_C + H_| > |ΔANPP_G_C + H_|	The decrease of ANPP_C_C + H_ due to forbs
ΔANPP_C_C + H_ < 0	|ΔANPP_S_C + H_| = |ΔANPP_G_C + H_| & |ΔANPP_S_C + H_| = |ΔANPP_F_C + H_|	The decrease of ANPP_C_C + H_ due to all three groups equally
ΔANPP_C_C + H_ = 0	|ΔANPP_S_C + H_| > |ΔANPP_G_C + H_| & |ΔANPP_S_C + H_| > |ΔANPP_F_C + H_|	No change of ANPP_C_C + H_ due to sedges
ΔANPP_C_C + H_ = 0	|ΔANPP_G_C + H_| > |ΔANPP_S_C + H_| & |ΔANPP_G_C + H_| > |ΔANPP_F_C + H_|	No change of ANPP_C_C + H_ due to graminoids
ΔANPP_C_C + H_ = 0	|ΔANPP_F_C + H_| > |ΔANPP_S_C + H_| & |ΔANPP_F_C + H_| > |ΔANPP_G_C + H_|	No change of ANPP_C_C + H_ due to forbs
ΔANPP_C_C + H_ = 0	|ΔANPP_S_C + H_| = |ΔANPP_G_C + H_| & |ΔANPP_S_C + H_| = |ΔANPP_F_C + H_|	No change of ANPP_C_C + H_ due to all three groups equally

Notes: ANPP_C_C+H_, aboveground net primary productivity of the plant community under the combined effects of climate change and human activities. ANPP_S_C+H_, ANPP_G_C+H_, ANPP_F_C+H_, same for sedges, graminoids, and forbs, respectively. Δ, change rate. |·|, absolute value. &, logical “and”. > , < , = indicate greater than, less than, or equal to, respectively.

## Results

**Fig 1:**
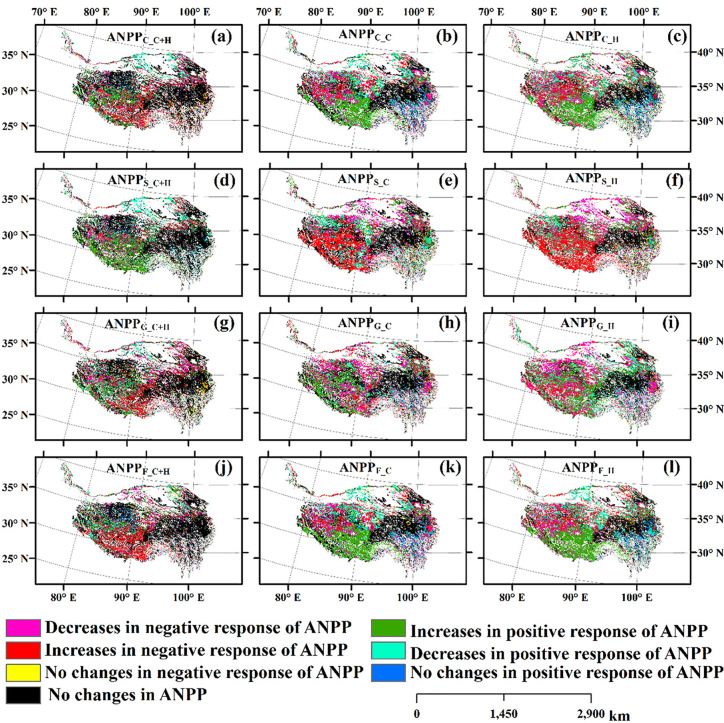
Spatial distribution for the changes in fresh aboveground net primary production (ANPP) of plant community (ANPP_C_), sedges (ANPP_S_), graminoids (ANPP_G_) and forbs (ANPP_F_) in 2000–2022. Note: The subscripts _C + H, _C, and _H denote the combined effects of climate change and human activities, the single effect of climate change, and the single effect of human activities, respectively.

The spatial averages RC_ANPP_C_C+H_, RC_ANPP_S_C+H_, RC_ANPP_G_C+H_ and RC_ANPP_F_C+H_ were –1.15%, 5.67%, –1.55%, and –2.26%, respectively. The spatially averaged negative change of ANPP_C_C+H_ (–0.13 per decade) and ANPP_F_C+H_ (–0.55 per decade) was increasing year by year, while the spatially averaged positive change of ANPP_S_C+H_ (0.19 per decade) was also increasing. By contrast, the spatially averaged negative change of ANPP_G_C+H_ (0.01 per decade) was decreasing year by year. The relative changes of ANPP were spatially heterogeneous (**Fig 1** and **S1 Fig**). For example, the positive responses of the ANPP_C_C+H_ in 13.25%, 7.42% and 2.56%, the ANPP_S_C+H_ in 23.65%, 9.55% and 3.33%, the ANPP_G_C+H_ in 10.37%, 8.79% and 0.98%, and the ANPP_F_C+H_ in 9.29%, 9.25% and 3.32% of the grassland areas were strengthening, weakening and remaining unchanged year by year, respectively (**Fig 1** and **S1 Fig**). In contrast, the negative responses of the ANPP_C_C+H_ in 9.16%, 15.03% and 2.28%, the ANPP_S_C+H_ in 7.10%, 7.35% and 0.42%, the ANPP_G_C+H_ in 10.49%, 15.48% and 3.69%, and the ANPP_F_C+H_ in 7.50%, 19.82% and 1.05% of the grassland areas were weakening, strengthening and remaining unchanged year by year, respectively (**Fig 1** and **S1 Fig**). In addition, the ANPP_C_C+H_, ANPP_S_C+H_, ANPP_G_C+H_, and ANPP_F_C+H_ did not change in 50.30%, 48.61%, 50.20% and 49.77% of the grassland areas, respectively (**Fig 1** and **S1 Fig**).

**Fig 2:**
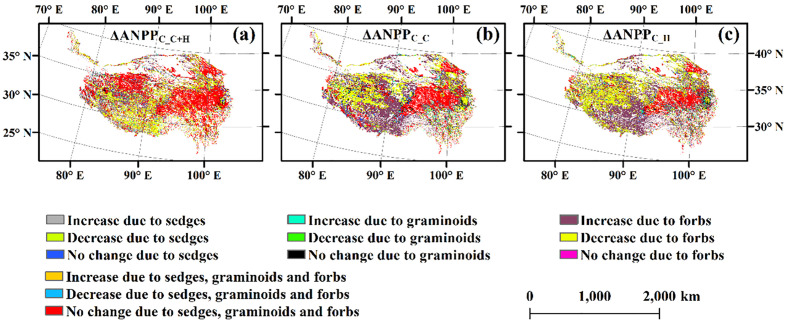
Spatial distribution for the relative contributions of changes in fresh aboveground net primary production (ANPP) of sedges, graminoids, and forbs to the change in ANPP of the plant community under different scenarios. Note: The subscripts _C + H, _C, and _H denote the combined effects of climate change and human activities, the single effect of climate change, and the single effect of human activities, respectively. ΔANPP_C_ represents the change rate of plant community ANPP.

The dominant influences of the changes in fresh ANPP of sedges, graminoids and forbs on the changes in fresh ANPP of plant community were spatially heterogeneous (**Fig 2**). For example, sedge fresh ANPP changes dominated the ANPP_C_C+H_, ANPP_C_C_ and ANPP_C_H_ changes by 18.27%, 8.28% and 12.47%, respectively; graminoid ANPP changes by 7.18%, 13.66% and 9.55%, respectively; forb ANPP changes by 35.43%, 54.40% and 61.85%, respectively; and the combined ANPP changes of all three by 39.12%, 23.65% and 16.13%, respectively (**Fig 2**). In addition, the increases in fresh ANPP_C_C+H_ of 9.14%, 1.40% and 12.40% of the grasslands were attributed to the increases in fresh ANPP_S_C+H_, ANPP_G_C+H_, and ANPP_F_C+H_ (**Fig 2**). Conversely, the decreases in fresh ANPP_C_C+H_ of 5.02%, 2.66% and 18.42% of the grasslands were attributed to the decreases in fresh ANPP_S_C+H_, ANPP_G_C+H_, and ANPP_F_C+H_, respectively (**Fig 2**).

**Fig 3:**
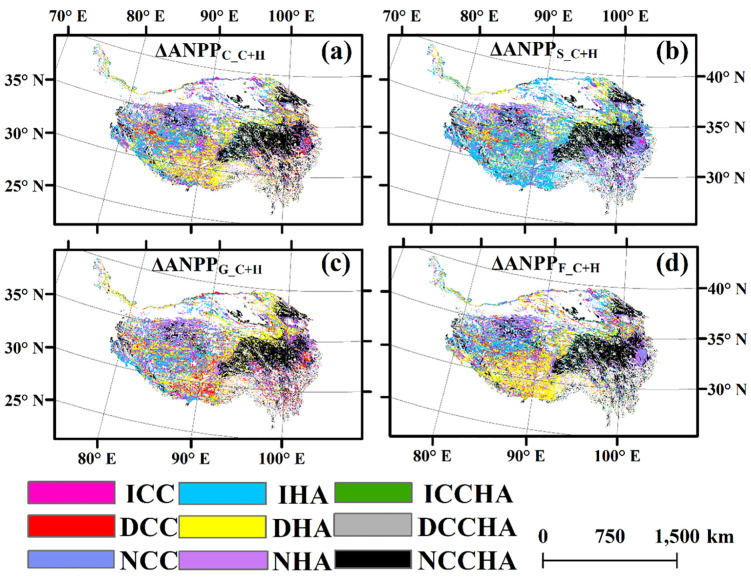
Spatial distribution of the relative contribution of climate change and human activities to the change in fresh aboveground net primary production (ANPP) under different scenarios for (a) plant community, (b) sedges, (c) graminoids, and (d) forbs. Note: The subscripts _C + H denote the combined effects of climate change and human activities. ICC: increases due to climate change; DCC: decreases due to climate change; NCC: no changes due to climate change; IHA: increases due to human activities; DHA: decreases due to human activities; NHA: no changes due to human activities; ICCHA: increases due to the combined effects of climate change and human activities; DCCHA: decreases due to the combined effects of climate change and human activities; NCCHA: no changes due to the combined effects of climate change and human activities.

The areas of grasslands where the fresh ANPP of plants increased, decreased and remained unchanged due to human activities were each larger than those caused by climate change (**Fig 3**). The proportions of grassland areas where the ΔANPP_C_C+H_, ΔANPP_S_C+H_, ΔANPP_G_C+H_ and ΔANPP_F_C+H_ were attributed to climate change were 20.79%, 17.02%, 24.08% and 18.01%, respectively, while those attributed to human activities were 54.26%, 60.73%, 52.36% and 56.87%, respectively (**Fig 3**). The remaining areas were jointly dominated by climate change and human activities (**Fig 3**).

**Fig 4:**
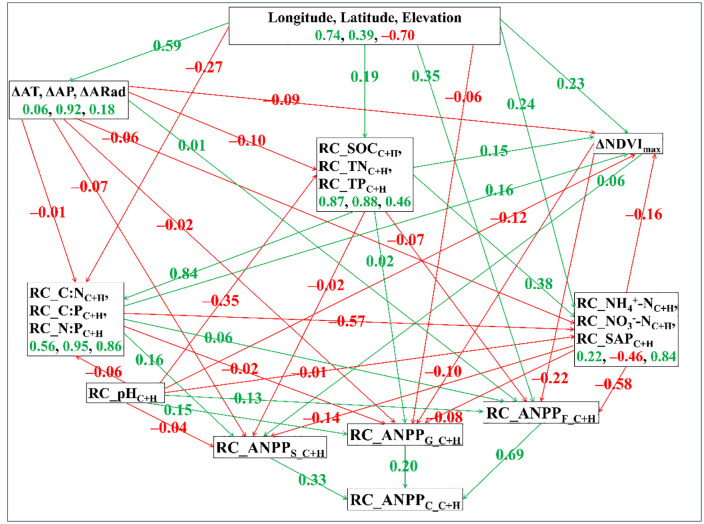
Structural equation model of fresh aboveground net primary production (ANPP) of plant community under the combined effects of climate change and human activities (ANPP_C_C + H_). Note: ΔAT, ΔAP, ΔARad and ΔNDVI_max_ indicate the change rate of annual temperature, annual precipitation, annual radiation and maximum normalized difference vegetation index, respectively. RC_SOC_C+H_, RC_TN_C+H_, RC_TP_C+H_, RC_C:N_C+H_, RC_C:P_C+H_, RC_N:P_C+H_, RC_NH_4_^+^-N_C+H_, RC_NO_3_^-^-N_C+H_, RC_SAP_C+H_, RC_pH_C+H_, RC_ANPP_S___C+H_, RC_ANPP_G___C+H_, RC_ANPP_F___C+H_ and RC_ANPP_C___C+H_ indicated the relative change of soil organic carbon, total nitrogen, total phosphorus, ratio of SOC to TN, ratio of SOC to TP, ratio of TN to TP, ammonium nitrogen, nitrate nitrogen, soil available phosphorus, pH, ANPP of sedges, ANPP of graminoids, ANPP of forbs, and ANPP of plant community under the combined effects of climate change and human activities, respectively.

Geographical location, climate change, changes in NDVI_max_, and changes in soil variables directly or indirectly affected the changes in fresh ANPP of plant community (**Fig 4** and **S2 Fig**). The direct impacts of climate change on the RC_ANPP_S_C+H_ and RC_ANPP_G_C+H_ were negative, whereas the direct impact of climate change on the RC_ANPP_F_C+H_ was positive (**Fig 4**). The ΔNDVI_max_ had a positive effect on the RC_ANPP_S_C+H_, but negative effects on the RC_ANPP_G_C+H_ and the RC_ANPP_F_C+H_ (**Fig 4**). Among these, the impact magnitude of ΔNDVI_max_ on the RC_ANPP_F_C+H_ was the greatest (**Fig 4**). The RC_SAP_C+H_ affected RC_ANPP_C_C+H_ more strongly than RC_NH_4_^+^-N_C+H_ and RC_NO_3_^-^-N_C+H_ (**Fig 4**). The impact direction of RC_NH_4_^+^-N_C+H_ on the RC_ANPP_C_C+H_ was completely opposite to that of RC_NO_3_^-^-N_C+H_ on the RC_ANPP_C_C+H_ (**Fig 4**).

## Discussion

The RC_NH_4_^+^-N_C+H_ and RC_NO_3_^-^-N_C+H_ were negatively and positively correlated with RC_ANPP_C_C+H_, respectively (**Fig 4**), reflecting the multiple action mechanisms of NH_4_^+^-N and NO_3_^-^-N on plants. NO₃ ⁻ -N is negatively charged, resulting in weak adsorption by soil and strong mobility [24]. It is easily absorbed efficiently by plant roots along with water movement, promoting the accumulation of aboveground biomass. In contrast, NH₄ ⁺ -N is positively charged and tends to be adsorbed by soil colloids, leading to uneven spatial distribution and low absorption efficiency [25]. In acidic soils, it combines with H⁺ to exacerbate acidification [26], further inhibiting plant growth. On the other hand, after being absorbed by the roots, NO₃ ⁻ -N is transported to the aboveground parts through the xylem. In mesophyll cells, it is catalytically converted to NH₄⁺ by nitrate reductase (NR) and nitrite reductase (NiR), and finally synthesized into amino acids [27]. This process works in synergy with photosynthesis, enhancing carbon assimilation efficiency by promoting chlorophyll synthesis and enzyme activity [28]. In contrast, the assimilation of NH₄ ⁺ -N relies on the GS-GOGAT cycle, which consumes large amounts of carbon skeletons and ATP [29]. Excessive accumulation triggers ammonium toxicity, inhibits photosynthetic assimilation capacity, and depletes energy reserves [30].

The area of grasslands where the change in fresh ANPP dominated by human activities was larger than that where it was dominated by climate change (**Fig 3**). Moreover, the proportion of the area where the change in grassland productivity was dominated by human activities was also greater than that reported in previous studies [19]. This finding may be related to the following mechanisms. Human activities directly alter surface cover through fence installation, ecological migration, and transportation construction [31,32]. Although such disturbances damage soil structure and organic matter in the short term (**Fig 4**, **S2 Fig**), they promote vegetation restoration through enclosure management in the long run [33]. In contrast, the indirect impacts of climate change on land use (such as climate warming expanding the upper limit of grazing elevation) are characterized by lag and uncertainty [34–38]. Livestock foraging exerts direct and high-intensity pressure on plant communities, and their selective grazing (preferring high protein and low fiber content) rapidly alters the composition of functional groups [39]. In contrast, the impacts of climate change are gradual, and plants can buffer short-term fluctuations through phenological adjustment [3]. Current intensive animal husbandry (such as breed improvement and high-density breeding) has expanded the depth of impact of human activities, resulting in its regulatory range on ANPP exceeding historical levels [19,40,41].

This functional group-specific response (Fig 3b-d and **S1d-l Fig**), as well as the geographical variability in their contributions to community productivity (**Fig 2**), is similar to some previous studies [4,11,42]. These phenomena are mainly driven by the following mechanisms. Similar to some previous studies [3,43], the degrees of closeness between the changes in the fresh ANPP of sedges, graminoids and forbs and the changes in climate, soil nutrients and soil pH differed, and even the directions also differed (**Fig 4**). Seeds of sedges and graminoids are primarily wind- or animal-dispersed, whereas forbs often rely on specific insects or small mammals for dispersal [44,45]. Global warming indirectly affects forb reproduction by altering pollinator insect distributions [46,47]. Overgrazing damages soil seed banks, inhibiting regeneration across functional groups [48]. Sedges and graminoids exhibit compensatory growth (increased tillering, enhanced photosynthetic capacity) under low-to-moderate grazing, whereas forbs are more vulnerable to soil degradation from intensive grazing and trampling [49,50]. Climate and anthropogenic activities indirectly regulate plant growth by altering soil/phyllosphere microbial community structures [51, 52]. Significant differences in symbiotic relationships between functional groups and microorganisms [50,53], drive divergent fresh ANPP responses [54–56]. Forbs expand rapidly in resource-rich areas, while in resource-limited regions, graminoids dominate fresh ANPP changes through efficient resource utilization and deep-rooted competition. Sedges maintain fresh ANPP dominance in low-temperature areas (such as alpine meadows), and warming promotes the growth of graminoids and forbs. Forbs have strong water-capturing capabilities in humid areas, while graminoids in arid regions enhance their ANPP contributions through deep root systems. Sedges prefer moist soils with high organic matter content, graminoids are adapted to loose and well-aerated substrates, and forbs dominate under specific nutrient conditions (e.g., high phosphorus levels). The foraging preferences of different herbivores for functional groups (e.g., livestock prefer graminoids with low fiber content) and their spatiotemporal activity patterns further amplify the spatial differentiation in ANPP contributions.

The acceleration of changes in fresh ANPP across different regions (i.e., the rate of change in ANPP itself) exhibits diversity (**Fig 1**). This diversity essentially arises from the spatial heterogeneity of five key dimensions: the rate of climate-driven changes, variations in the intensity of human activities, resource limitation thresholds, the direction of biological feedbacks, and the intensity of multi-factor interactions. Climate serves as the fundamental driver of fresh ANPP (**Fig 4**), but the intensity and trends of changes in climatic factors vary significantly across regions [9], directly leading to differences in the acceleration of fresh ANPP changes. Changes in the intensity of human activities (such as grazing), whether increasing, decreasing, or shifting direction, directly alter the rate of fresh ANPP change (**S2 Fig**), and such “intensity variations” show substantial regional differences. Fresh ANPP growth depends on resources such as water and nutrients (nitrogen, phosphorus, etc.) (**Fig 4**). When the “matching degree” between resource supply and vegetation demand changes [57], it triggers a turning point in acceleration, with significant regional variations in the thresholds of this process. Changes in vegetation itself (e.g., coverage, species composition) form feedbacks through “resource capture and cycling”. The “positive/negative direction” and “intensity changes” of these feedbacks alter fresh ANPP acceleration, with marked regional differences.

While our analysis revealed spatially heterogeneous patterns in the intensification or weakening of fresh ANPP trends (**Fig 1**), we acknowledge inherent difficulty in precisely quantifying the rate of change of these trends (ΔΔANPP). The relatively short temporal span of our dataset (23 years) and the natural interannual variability in grassland productivity data introduce significant uncertainty into estimates of second-order derivatives. Short time series are particularly sensitive to the influence of extreme years or short-term fluctuations, which can amplify noise and potentially obscure underlying long-term accelerations or decelerations. Therefore, the ΔΔANPP values presented in **Table 1** and **Fig 1** should be interpreted primarily as qualitative indicators of directional changes in trend magnitude (i.e., whether a positive or negative trend was generally strengthening or weakening over the study period) rather than precise quantitative measures of acceleration. The spatial patterns indicating where intensification or weakening occurred (**Fig 1**) are likely more robust and ecologically informative than the exact numerical values of ΔΔANPP itself. These patterns, when viewed together with the dominant drivers identified (e.g., human activities dominating larger areas, **Fig 3**; the differential roles of functional groups, **Fig 2**; and the influence of soil N forms, **Fig 4**), provide valuable insights into the dynamics and potential vulnerability of the Tibetan grassland ecosystems under changing environmental pressures. Future research based on longer-term monitoring will be crucial to confirm the persistence of these observed trends dynamics and refine estimates of their rates of change.

To directly address the core issue of spatiotemporal decoupling, this study employed a key methodological strategy: model construction relied exclusively on annually varying dynamic drivers (temperature, precipitation, radiation, NDVI_max_), completely excluding static covariates (e.g., soil properties or elevation) as inputs. This design forces the model to resolve spatiotemporal signals through the dynamic drivers themselves. Model validation was conducted through strict randomization partitioning of all site-year observations, ensuring simultaneous evaluation of predictive performance on unseen locations and unseen years. The dataset encompasses three main grassland types (reflecting spatial heterogeneity) with a temporal depth of ≥6 years, establishing a foundation for assessing the model’s generalization capability across novel spatiotemporal combinations. Although the aforementioned methods effectively address the core concern, the following residual uncertainties must be clarified. The model utilizes annually aggregated driver data, yet key ecological processes governing fresh ANPP (e.g., timing of snowmelt, onset of the growing season, occurrence of extreme events such as late frosts/short-term droughts, intra-seasonal grazing patterns) operate at finer temporal scales. Annual aggregation inevitably smooths critical intra-annual dynamics, potentially masking their nuanced effects on productivity, particularly for different plant functional groups with varying phenological sensitivities. Although excluding static variables forces the model to learn dynamics, the sensitivity of fresh ANPP to interannual fluctuations in climatic and human activity proxies still varies spatially. This stems from unmodeled factors (e.g., inherent soil fertility, microtopography, historical legacy effects). While model validation assessed overall spatiotemporal predictive performance, it cannot guarantee uniform accuracy in capturing the magnitude of temporal change across all heterogeneous grassland types. Compared to the model’s generalized relationships, sites with inherent low nutrient availability or poor water retention capacity may exhibit dampened or amplified responses to climatic drivers. The identified dominant role of forb ANPP changes (**Fig 2**) and the core function of nitrate/ammonium dynamics (**Fig 4**) emerge from landscape-scale patterns. While statistically robust at this scale, they may not be directly extrapolated to mechanistic understanding at the patch or individual plant level. Manipulative experiments targeting specific factors (e.g., N form addition, selective grazing exclusion) remain crucial for validating the physio-ecological mechanisms underpinning the observed large-scale correlations. Consequently, estimates of the absolute rates of change and the precise areal proportions dominated by climate vs. human activities (**Fig 3**) should be regarded as the best possible spatially explicit reconstruction given the model structure and input data, with their quantitative accuracy constrained by the aforementioned uncertainties. Nevertheless, the core contribution of this study lies in revealing the following key new insights. The spatial differentiation pattern of change trends (intensification/weakening) (**Fig 1**), the differential responses of plant functional groups, the pan-plateau dominance of human activities relative to climate change, the contrasting ecological roles of soil nitrogen forms (nitrate-N vs. ammonium-N). These findings provide important evidence for understanding the ecosystem functioning of Tibetan alpine grasslands under global change pressures. Future research needs to integrate data with higher temporal resolution, manipulative experiments across environmental gradients, and process-based models to refine the quantitative assessments presented herein and elucidate their underlying mechanisms.

## Conclusions

This study aimed at the deficiencies in previous studies on the fresh aboveground net primary productivity (ANPP), the ANPP of functional groups, and the intensity of change trends. Taking the alpine grasslands on the Qinghai-Xizang Plateau from 2000 to 2022 as the research objects, it thoroughly analyzed the change patterns of the fresh ANPP of the plant community and three functional groups (i.e., sedges, graminoids, and forbs), as well as their correlations with independent variables such as soil carbon, nitrogen, and phosphorus, striving to reveal the change rules and internal mechanisms of fresh ANPP under the dual influences of climate change and human activities. The relevant research conclusions are of great significance both scientifically and practically. Scientifically, they provide detailed and reliable bases for further exploring the functions of alpine grassland ecosystems and their dynamic change mechanisms. Practically, they can build a solid foundation for formulating precise and effective ecological protection and management strategies.

Regarding the change trends of fresh ANPP, the plant community and each functional group showed different performances. Under the combined effects of climate change and human activities, the fresh ANPP of the community generally showed a negative growth, with the spatially averaged relative change rate being –1.15%. Among them, the fresh ANPP of sedges increased by 5.67%, while the fresh ANPP of graminoids and forbs decreased by 1.55% and 2.26%, respectively. This phenomenon reveals a broader ecological principle: alpine ecosystems are undergoing functional restructuring under global change, rather than simply experiencing an overall decline. Such restructuring triggers significant shifts in key ecological processes. The expansion of sedges, with their dense root systems, might enhance soil carbon sequestration capacity, while the reduction of forbs could disrupt trophic networks. In high-altitude regions sensitive to global change, the differential dynamics of functional groups serve as a core indicator for predicting ecosystem resilience. As an iconic site for studying climate–ecosystem interactions, this pattern on the Qinghai-Xizang Plateau not only demonstrates the mechanisms of species filtering and community restructuring under extreme environmental stress but also provides a typical example for understanding the non-linear responses of high-altitude biomes worldwide.

The positive responses in some regions showed increasing or decreasing trends over time, and the negative responses showed similar changes, while in other regions the changes were not significant. This scientific discovery warns that regional differences should be considered when formulating ecological management strategies. For regions with an increasing positive response, especially those where the fresh ANPP of sedges and graminoids increased simultaneously, measures can be taken to further promote this positive change. For regions with a decreasing positive response, it is necessary to analyze the reasons, which might be overgrazing or the emergence of new disturbing factors, so as to adjust management strategies in a timely manner (e.g., reducing grazing intensity and controlling alien species invasion). For regions with an increasing negative response, large-scale ecological restoration projects may be required, such as vegetation reconstruction and soil improvement. However, for regions with a decreasing negative response, some relatively mild measures (e.g., fencing for natural restoration) can be adopted to help the ecosystem recover.

From the perspective of the dominance of influencing factors, the area where human activities dominated the changes in grassland fresh ANPP was larger than that of climate change. In this regard, managers could formulate targeted grazing management measures to effectively reduce the negative impacts of human activities on grassland ecosystems and enhance the positive impacts, thereby helping grassland ecosystems achieve healthy and stable development.

The contributions of fresh ANPP of different functional groups to the changes in fresh ANPP of the plant community showed geographical differences. Under the combined effects of climate change and human activities, the area of grassland jointly regulated by the three functional groups accounted for the largest proportion, and the areas dominated by fresh ANPP of forbs, sedges, and graminoids decreased in that order. This difference is closely related to plant preferences for temperature and humidity, soil conditions, and the feeding preferences of herbivores. Based on a clear understanding of this situation, the management of grassland resources can be made more scientific and reasonable. For example, in specific regions, the utilization methods of grasslands could be carefully planned according to the characteristics of the dominant functional groups to achieve optimal resource allocation, thereby enhancing grassland productivity and fully exerting the service functions of grassland ecosystems.

In addition, the impacts of nitrate nitrogen and ammonium nitrogen in soils on the fresh ANPP of the community are also different. When carrying out ecological restoration of alpine grasslands, considering the differences between nitrate nitrogen and ammonium nitrogen is helpful to optimize restoration strategies. For example, in some grasslands degraded due to nitrogen loss, if the loss of nitrate nitrogen seriously affects plant growth, measures can be taken to reduce nitrate nitrogen leaching, such as improving soil structure or increasing soil organic matter to enhance the soil’s ability to retain nitrate nitrogen. Meanwhile, according to plant requirements for different forms of nitrogen, suitable plant species can be selected for reseeding to improve the success rate of ecological restoration.

## Supporting information

S1 Fig The relative change (RC_) of aboveground net primary production (ANPP) of plant community (ANPP_C_), sedges (ANPP_S_), graminoids (ANPP_G_) and forbs (ANPP_F_) in 2000–2022. Note: __C+H_, __C_ and __H_ indicated the scenes of the combined effects of climate change and human activities, the single effect of climate change, and the single effect of human activities, respectively.
(DOCX)

S2 Fig Structural equation model of aboveground net primary production (ANPP) of plant community under the sole effect of climate change (ANPP_C_C_) and human activities (ANPP_C_H_), respectively. Note: ΔAT, ΔAP and ΔARad indicated the change rate of annual temperature, annual precipitation and annual radiation, respectively. RC_ indicated relative change. __C_ and __H_ indicated the scenes of the sole effect of climate change and the sole effect of human activities, respectively. SOC, soil organic carbon; TN, total nitrogen; TP, total phosphorus; C:N, ratio of SOC to TN; C:P, ratio of SOC to TP; N:P, ratio of TN to TP; NH_4_^+^-N, ammonium nitrogen; NO_3_^-^-N, nitrate nitrogen; SAP: soil available phosphorus; ANPP_S_, aboveground net primary production (ANPP) of sedges; ANPP_G_, ANPP of graminoids; ANPP_F_, ANPP of forbs; ANPP_C_, ANPP of plant community.
(DOCX)
