# Supplementary material for: Fresh aboveground net primary productivity of Tibetan grasslands: Responses of different plant functional groups to climate change and human activities and implications for ecosystem management
Source: PLoS One. 2026 Jun 10;21(6):e0349705. doi: 10.1371/journal.pone.0349705 (PMC13252736; doi:10.1371/journal.pone.0349705)
Supplement: S1 Fig — Note: _C+H, _C and _H indicated the scenes of the combined effects of climate change and human activities, the single effect of climate change, and the single effect of human activities, respectively. (DOCX) [file pone.0349705.s001.docx]

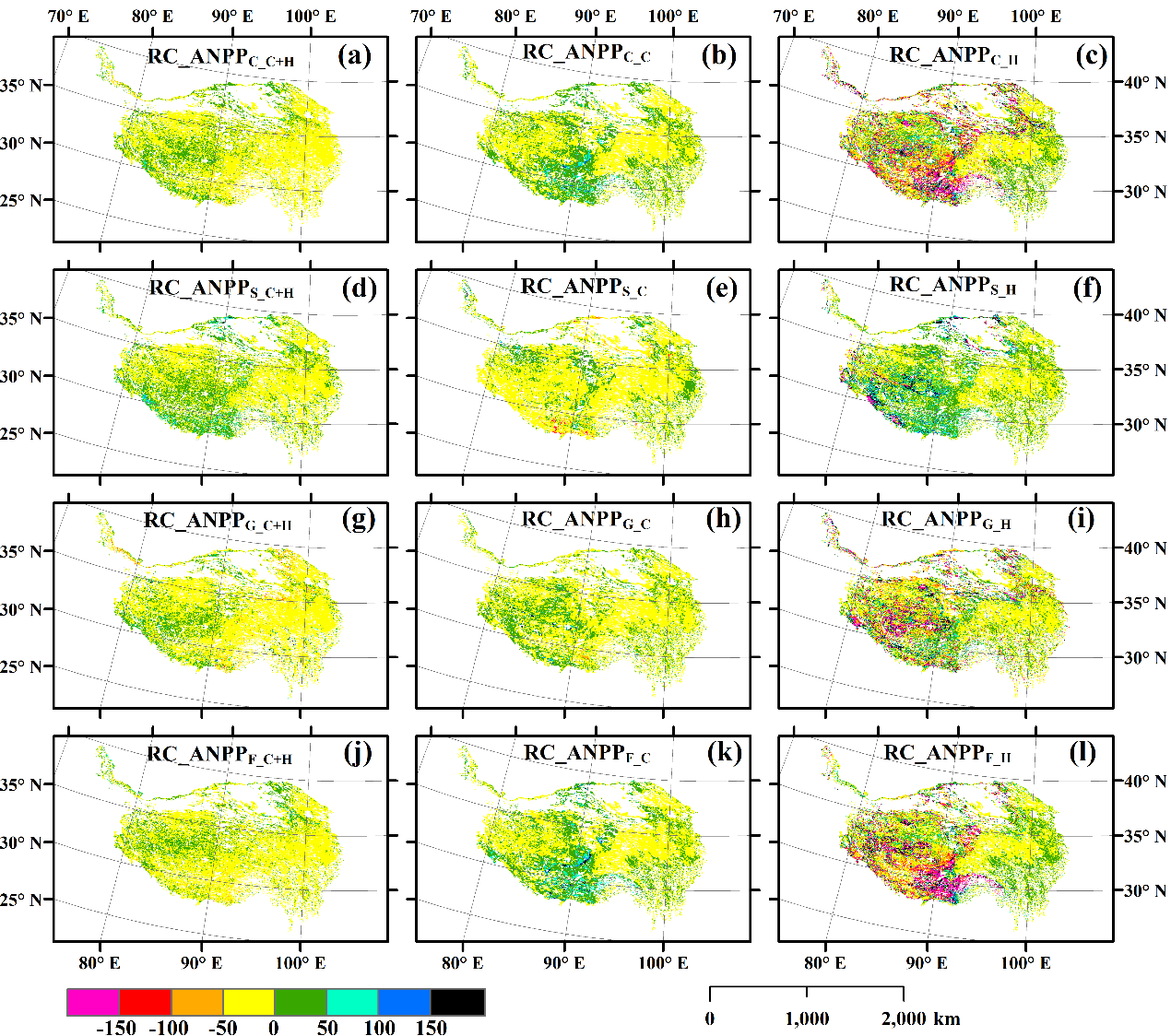
**S1 Fig**. The relative change (RC_) of aboveground net primary production (ANPP) of plant community (ANPP_C_), sedges (ANPP_S_), graminoids (ANPP_G_) and forbs (ANPP_F_) in 2000–2022.

Note:__C+H_, __C_ and __H_ indicated the scenes of the combined effects of climate change and human activities, the single effect of climate change, and the single effect of human activities, respectively.
