# Supplementary material for: Fresh aboveground net primary productivity of Tibetan grasslands: Responses of different plant functional groups to climate change and human activities and implications for ecosystem management
Source: PLoS One. 2026 Jun 10;21(6):e0349705. doi: 10.1371/journal.pone.0349705 (PMC13252736; doi:10.1371/journal.pone.0349705)
Supplement: S2 Fig — Note: ΔAT, ΔAP and ΔARad indicated the change rate of annual temperature, annual precipitation and annual radiation, respectively. RC_ indicated relative change. _C and _H indicated the scenes of the sole effect of climate change and the sole effect of human activities, respectively. SOC, soil organic carbon; TN, total nitrogen; TP, total phosphorus; C:N, ratio of SOC to TN; C:P, ratio of SOC to TP; N:P, ratio of TN to TP; NH4+-N, ammonium nitrogen; NO3--N, nitrate nitrogen; SAP: soil available phosphorus; ANPPS, aboveground net primary production (ANPP) of sedges; ANPPG, ANPP of graminoids; ANPPF, ANPP of forbs; ANPPC, ANPP of plant community. (DOCX) [file pone.0349705.s002.docx]

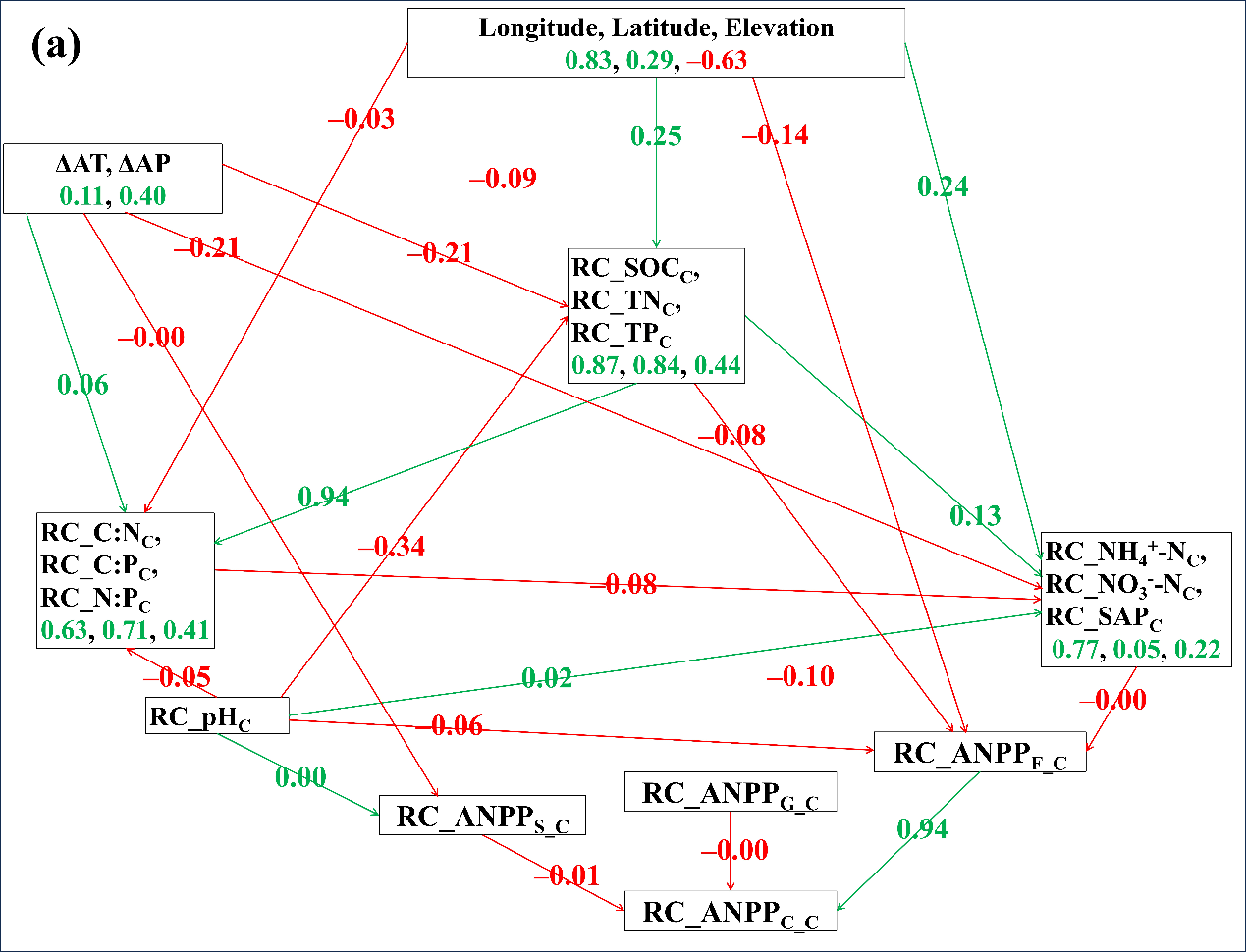


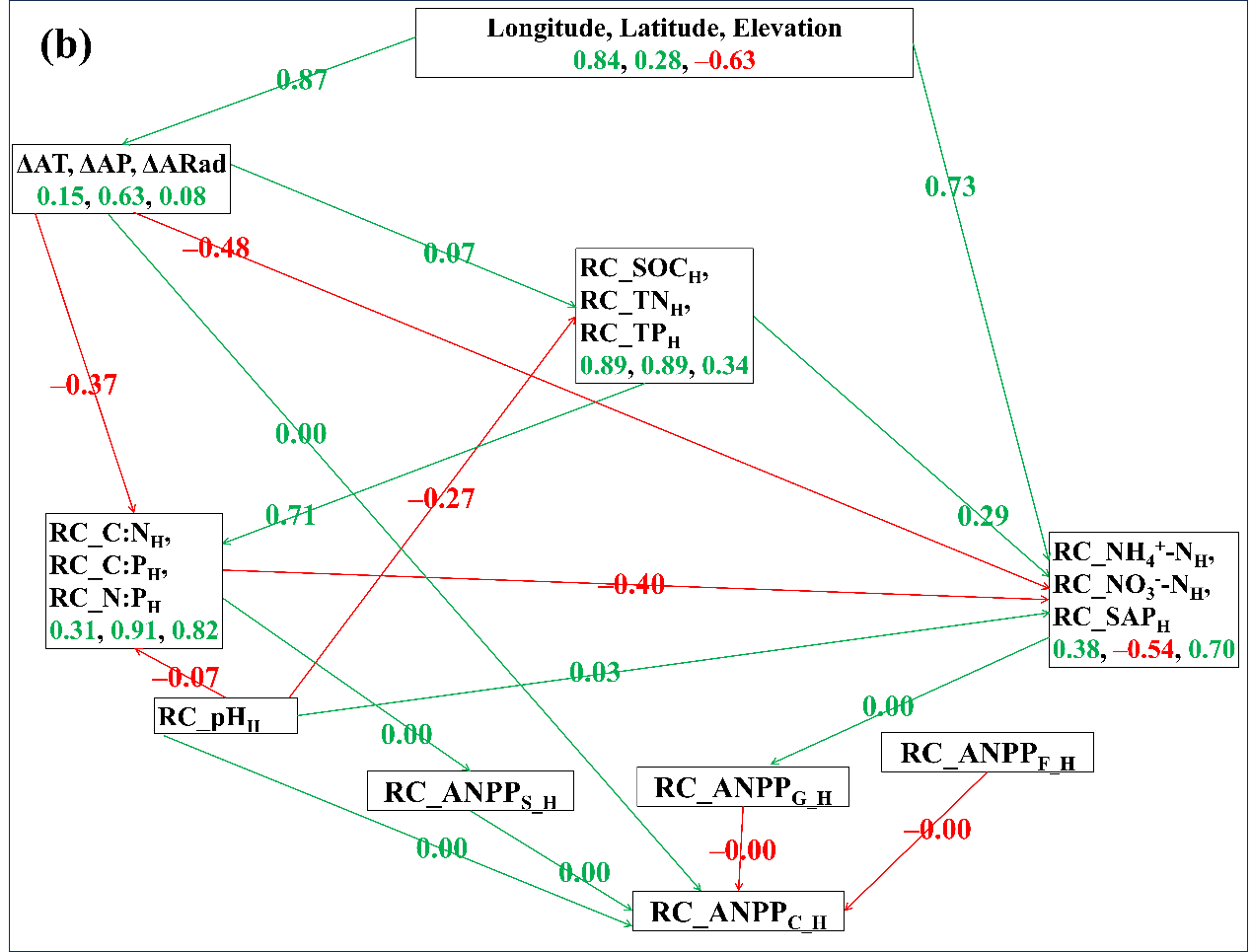


**S2 Fig**. Structural equation model of aboveground net primary production (ANPP) of plant community under the scene of the sole effect of climate change (ANPP_C_C_) and human activities (ANPP_C_H_), respectively. ΔAT, ΔAP and ΔARad indicated the change rate of annual temperature, annual precipitation and annual radiation, respectively. RC_ indicated relative change.

Note:__C_ and __H_ indicated the scenes of the sole effect of climate change and the sole effect of human activities, respectively. SOC, soil organic carbon; TN, total nitrogen; TP, total phosphorus; C:N, ratio of SOC to TN; C:P, ratio of SOC to TP; N:P, ratio of TN to TP; NH_4_^+^-N, ammonium nitrogen; NH_3_^-^-N, nitrate nitrogen; SAP: soil available phosphorus; ANPP_S_, aboveground net primary production (ANPP) of sedges; ANPP_G_, ANPP of graminoids; ANPP_F_, ANPP of forbs; ANPP_C_, ANPP of plant community.
